# Five-year outcomes of selective laser trabeculoplasty: A retrospective study

**DOI:** 10.3389/fmed.2022.1039195

**Published:** 2023-01-12

**Authors:** David L. Swain, Babak Eliassi-Rad

**Affiliations:** ^1^Department of Ophthalmology, Boston University School of Medicine, Boston, MA, United States; ^2^Department of Anatomy and Neurobiology, Boston University School of Medicine, Boston, MA, United States

**Keywords:** selective laser trabeculoplasty, intraocular pressure, glaucoma, retrospective chart review, optical coherence tomography, retinal nerve fiber thickness

## Abstract

**Introduction:**

Studies have shown the efficacy of selective laser trabeculoplasty (SLT) to lower intraocular pressure (IOP) as adjuvant therapy during short-term follow-up. However, few studies have assessed the long-term efficacy of SLT on preventing worsening Humphrey visual field (HVF) parameters and thinning of the retinal nerve fiber layer (RNFL) with continued medical therapy.

**Methods:**

A retrospective chart review was conducted of 51 eyes of 39 patients with glaucoma treated with SLT at Boston Medical Center between 2012 and 2016 with 3- and 5-year follow-up. Outcome measures included IOP, visual acuity, number of glaucoma medications, number of months to subsequent surgical intervention. HVF outcome measures included mean deviation (MD) and pattern standard deviation (PSD). Optical coherence tomography (OCT) outcome measures included RNFL mean thickness, and superior and inferior thicknesses.

**Results:**

Twenty-five eyes received subsequent surgical intervention (mean time to intervention = 33.6 ± 20.0 months). In the eyes that did not receive another intervention, mean IOP was significantly decreased by 3.2 and 3.5 mmHg at 3- and 5-year after SLT, respectively. Mean number of glaucoma medications was significantly increased at 5-year (2.7 ± 1.6; *P* = 0.04), compared to pre-SLT (2.0 ± 1.1). Mean HVF MD was significantly higher at 5-year (−7.64 ± 6.57 dB) compared to pre-SLT (−5.61 ± 3.90 dB). Mean PSD significantly increased at 3-year (5.30 ± 2.91 dB) and 5-year (6.84 ± 2.62 dB), compared to pre-SLT (4.63 ± 2.70 dB; *P* = 0.04 and ≤0.01, respectively). On OCT, inferior quadrant RNFL thickness decreased significantly at 5-year (88.5 ± 19.3 μm), compared to pre-SLT (94.0 ± 23.2 μm).

**Discussion:**

Although 51% of eyes had IOP controlled at 5-year post-SLT, mean number of glaucoma medications was significantly higher. Also, there was progression of MD and PSD on HVF and inferior quadrant thinning on OCT at 5-year. We found a significant association between age at SLT and risk of subsequent surgical intervention over 5-year follow-up. Our study adds to our understanding of long-term outcomes of adjuvant SLT for glaucoma patients receiving medical therapy.

## 1. Introduction

Glaucoma is one of the leading causes of irreversible blindness worldwide with an estimated prevalence of 79.6 million in 2020 with 74% of cases being primary open-angle glaucoma (POAG) (1, 2). Glaucoma is characterized by degeneration of the retinal ganglion cells and their axons (3). The only modifiable risk factor for glaucoma is intraocular pressure (IOP) (4). Current first-line intervention to lower IOP is medications (i.e., eyedrops); however, laser procedures, including selective laser trabeculoplasty (SLT), have been shown to be effective at lowering IOP in the short-term follow-up period and are most commonly used for adjuvant and first-line therapy (5).

Selective laser trabeculoplasty utilizes a Q-switched, frequency-doubled Nd:YAG laser to target pigmented cells of the trabecular meshwork selectively that leads to acute changes in the anterior angle ultrastructure, including disruption of the uveal trabecular beams (6, 7). SLT has also been shown to lead to increased activity of metalloproteinases that remodel the juxtacanalicular connective tissue matrix (8) and increased permeability of Schlemm’s canal endothelial cells (9). Both of these changes lead to increased aqueous humor outflow and lower IOP to prevent progression of glaucomatous damage.

Many studies have investigated the clinical value of first line or adjuvant SLT in the short-term period following treatment and have found that SLT is effective at lowering IOP in patients with glaucoma (10–17). Many studies of adjuvant SLT therapy have reported decreases in IOP of ∼29% up to 4 years following treatment (13). However, only a few studies have explored the long-term (>4 years) outcomes of SLT on lowering IOP and even fewer have examined functional analyses of vision, including Humphrey visual fields (HVF) and retinal nerve fiber layer (RNFL).

Although SLT has been shown to be an effective method in lowering IOP in patients with glaucoma, there is not a consensus and not enough literature to assess whether SLT works in all clinical settings and whether its effects on IOP are long-lasting and prevent further loss of function on HVF and OCT exams. Therefore, in this study, we investigated the long-term outcomes of SLT after 3- and 5-year on patient parameters including IOP, visual acuity (VA), HVF parameters, and RNFL thickness on OCT using a retrospective chart review of glaucoma patients with continued medical therapy at Boston Medical Center.

## 2. Materials and methods

### 2.1. Study design

**FIGURE 1:**
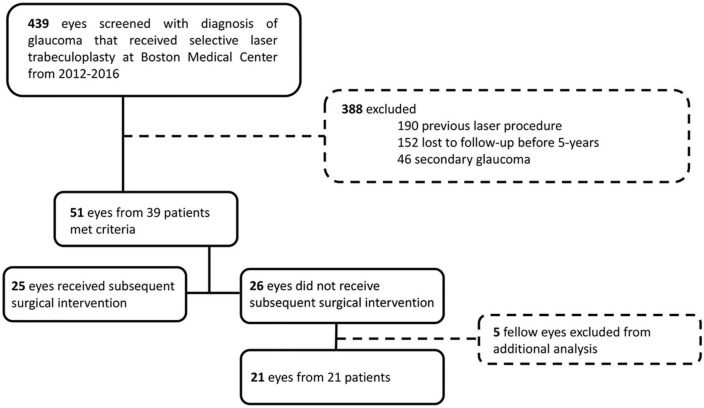
CONSORT diagram for retrospective chart review. We retrospectively screened 439 charts of eyes with diagnosis of glaucoma that received SLT at Boston Medical Center from 2012 to 2016. We excluded 388 eyes that had a previous laser procedure, were lost to follow-up within 5-year, or had secondary open-angle glaucoma or angle-closure glaucoma. We included 51 eyes, who had sufficient 5-year follow-up, of which 25 received subsequent surgical intervention and 26 did not. For analysis of the outcome measures of the patients that did not receive subsequent surgical intervention, we excluded fellow eyes to only analyze one eye per patient.

A single-center retrospective review evaluated patients with glaucoma who underwent SLT from 2012 to 2016 at Boston Medical Center, Boston, MA. This study adhered to tenets of the Declaration of Helsinki and was approved by the Institutional Review Board of Boston University (IRB: H-39121). Four hundred and thirty-nine charts were examined to identify patients with a diagnosis of glaucoma (POAG, borderline, suspect with ocular hypertension, normal tension glaucoma, or uveitic) with follow-up within 6 months of 3- and 5-year following SLT (Figure 1). We excluded patients with other forms of secondary open-angle glaucoma and angle-closure glaucoma, and we excluded patients who had received previous laser procedures, including argon laser trabeculoplasty or SLTs performed before treatment at Boston Medical Center. Fifty-one eyes of 39 patients (20 male, 19 female) followed for a mean of 68.2 ± 12.4 months following SLT at Boston Medical Center were identified and used in this study (Figure 1). Data were recorded from pre-SLT and at 3- and 5-year follow-ups. Data included age at SLT, gender, self-identified ethnicity, IOP, VA (reported as a decimal), number of glaucoma medications, HVF mean deviation (MD) and pattern standard deviation (PSD), OCT RNFL mean thickness, and superior and inferior thicknesses.

### 2.2. Selective laser trabeculoplasty and functional assessments

Lower intraocular pressure was measured before SLT, immediately after SLT, 6 weeks after SLT, and then typically every 6–12 months. SLTs were performed at Boston Medical Center from 2012 to 2016 using a Lumenis Selecta^®^ Trio™ (Lumenis Inc., CA, USA), which is a Q-switched Nd:YAG laser. Laser wavelength was 532 nm with a spot size of 400 μm and pulse duration of 3 ns. Energy levels ranged from 0.4 to 1.2 mJ per pulse. All eyes received 50–62 adjacent, non-overlapping pulses to 180° of the trabecular meshwork, except one eye which received 100 pulses to 360° of meshwork. If the eyes received a second SLT, the previously untreated 180° of trabecular meshwork were treated. Factors that were weighed between physician and patient when assessing need for subsequent surgical intervention included IOP not at target, patient adherence to medications, patient on maximally tolerated medical treatment, risk of surgical complications, patient preference for or against surgery.

For functional assessments, HVF was performed with a Humphrey Field Analyzer 750i (Carl Zeiss Meditec, Inc., Dublin, CA, USA), and RNFL measurements were taken using a Cirrus HD-OCT 5000 (Carl Zeiss Meditec, Inc., Dublin, CA, USA).

### 2.3. Statistical analysis

All statistical analyses were performed using R Statistical Computing Package (v3,5,1l R Foundation for Statistical Computing, Vienna, Austria). All data are summarized as means ± SD. VA is reported as a decimal. For survival analysis, we performed a multilevel Cox proportional hazards regression analysis, with clustering by patient to account for the potential intraclass correlation from including both eyes for some patients, to calculate the survival probability of patients that received a subsequent surgical intervention over time in months during the follow-up period. For each eye, the amount of time (months) was calculated from SLT treatment to date of subsequent intervention, and eyes that did not receive subsequent intervention were censored at timepoint of last follow-up. This multilevel model with clustering also estimated hazard ratios for risk of receiving subsequent surgical procedure during the 5-year follow-up, while adjusting for confounding factors, including fellow eyes, age, sex, and pre-SLT IOP. In this model, we used a central corneal thickness (CCT) value of <555 μm, based on previous studies (18). Lastly, using the eyes that did not receive further surgical intervention following SLT to control IOP, we excluded fellow eyes, only including one eye per patient. We excluded fellow eyes by using a random number generator to randomly select the right or left eye to exclude. For these eyes, we examined differences in mean IOP, number of glaucoma medications, HVF MD and PSD, and OCT RNFL thickness using paired Student’s *t-*tests to compare the means at 3-year or 5-year to baseline values. A value of *P* < 0.05 was considered significant.

## 3. Results

Patient demographics are summarized in Table 1. The mean age at SLT treatment was 69.4 ± 8.0-year, and 69.8 ± 7.8 years for males and 69.0 ± 8.3 years for females. Of these patients, 82.3% self-identified as black or African American. Most eyes were diagnosed with POAG (40/51, 78.4%) or normal tension glaucoma (NTG) (5/51, 9.8%).

**TABLE 1:** Baseline demographic and clinical characteristics of eyes.

Characteristic	Mean or count
Age at SLT (years)	69.4 ± 8.0
**Gender**	
Male	25 (49.0%)
Female	26 (51.0%)
**Self-reported ethnicity**	
Black or African American	42 (82.3%)
Hispanic or Latino	3 (5.9%)
White	4 (7.8%)
Declined	2 (3.9%)
**Diagnosis**	
POAG	40 (78.4%)
Borderline	3 (5.9%)
Suspect	1 (2.0%)
NTG	5 (9.8%)
Uveitic	2 (3.9%)

POAG, primary open-angle glaucoma; NTG, normal tension glaucoma.

### 3.1. Time to subsequent glaucoma surgical intervention

**FIGURE 2:**
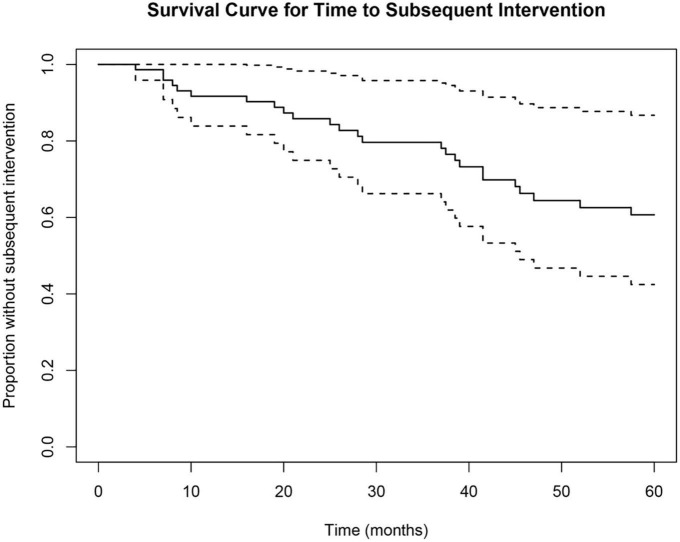
Cox proportional hazard survival curve for time to subsequent surgical intervention. Survival analysis showed a 79.6% survival probability at 36 months and a 60.7% survival probability at 60 months. Dotted lines represent upper and lower 95% confidence intervals.

Time to subsequent glaucoma surgical intervention was calculated for each eye from the SLT date. Of the 51 eyes, 25 (49%) received subsequent surgical intervention to lower IOP, and 26 (51%) did not receive further surgical intervention. Survival probability calculated by multilevel Cox proportional hazard model analysis was 79.6% at 36 months (3-year) and 60.7% at 60 months (5-year) (Figure 2). Of the eyes that received subsequent surgical intervention, 52% (13/25) underwent a repeat SLT. The counts of each type of first subsequent surgical intervention over the 5-year follow-up period are summarized in Table 2. Of the 25 eyes that received a subsequent surgical intervention, seven of them (28%) received more than one intervention (six eyes received two interventions; and one eye received three interventions total) within the 5-year follow-up period. None of the eyes received more than one additional SLT.

**TABLE 2:** Counts of subsequent surgical interventions.

Type of surgical intervention	Count
None	26
Repeat SLT	13
Ahmed valve implant	5
Trabeculectomy	3
MicroPulse™	2
CE	2
Total	51

SLT, selective laser trabeculoplasty; CE, cataract extraction.

### 3.2. Factors influencing time to subsequent surgical procedure

To identify factors that potentially contributed to receiving a subsequent surgical intervention within the 5-year follow-up period, we calculated hazard ratios of certain characteristics (Table 3). We found that central corneal thickness < 555 μm, female gender, and pre-SLT IOP, and left eye (OS) were not significantly associated with increased risk of subsequent surgical procedure over the 5-year follow-up period (all *P* > 0.05; Table 3). However, we found that higher patient age at SLT was significantly associated with an increased risk of receiving a subsequent surgical procedure (*P* = 0.04) (Table 3).

**TABLE 3:** Summary of hazard ratio analysis.

Characteristic	Hazard ratio over 5-year	95% confidence interval	*P*-value
Age at SLT	1.05	[1.01, 1.09]	**0.04**
CCT*	1.01	[0.99, 1.01]	0.24
Female gender	1.79	[0.70, 4.54]	0.22
Pre-SLT IOP	1.02	[0.91, 1.14]	0.76
OS	0.92	[0.49, 1.74]	0.80

CCT, central corneal thickness; OS, left eye. *Forty-one eyes were used for this analysis that had available CCT data pre-SLT. All other hazard ratios were determined using all 51 eyes included in this study with clustering among patients. Bold values indicate the *p*-value <0.05.

### 3.3. IOP, VA, and number of glaucoma medications

We further examined the eyes that did not receive a subsequent surgical intervention within the 5-year follow-up period and only used one eye from each patient (*n* = 21) in order to examine the effect of SLT alone with continued medical therapy. The outcome measures for these 21 eyes were analyzed to determine the effects of SLT provided over 5-year (Table 4). Mean IOP was significantly reduced at 3-year (14.8 ± 2.8 mmHg) and 5-year (14.5 ± 3.3 mmHg), compared to pre-SLT IOP (18.0 ± 3.7 mmHg; paired Student’s *t*-test; both *P* ≤ 0.01). VA (reported as a decimal) was not significantly different at 3-year (0.8 ± 0.2; *P* = 0.84) or 5-year (0.8 ± 0.2; *P* = 0.74), compared to pre-SLT VA (0.8 ± 0.2; paired Student’s *t*-tests). Number of glaucoma medications was significantly higher at 5-year (2.7 ± 1.6) compared to pre-SLT (2.0 ± 1.1; *P* = 0.04), but it was not different at 3-year (2.5 ± 1.3), compared to pre-SLT (*P* = 0.07) (Table 4).

**TABLE 4:** Outcome measures in eyes that did not receive subsequent surgical intervention.

		Mean*	SD	*P*-value^†^
IOP (mmHg)	Pre-SLT	18.0	3.7	–
	3-year	14.8	2.8	**≤0.01**
	5-year	14.5	3.3	**≤0.01**
VA	Pre-SLT	0.8	0.2	–
	3-year	0.8	0.2	0.84
	5-year	0.8	0.2	0.74
# Glaucoma medications	Pre-SLT	2.0	1.1	–
	3-year	2.5	1.3	0.07
	5-year	2.7	1.6	**0.04**

*Twenty-one eyes that did not receive subsequent surgical intervention in the 5-year follow-up period were used in this analysis. Only one eye from each patient was used in this analysis. ^†^*P*-values refer to comparison with pre-SLT values. Bold values indicate the *p*-value <0.05.

### 3.4. Humphrey visual field parameters

For the eyes that did not receive a surgical intervention in the follow-up period that had complete HVF data, we used only one eye from each patient (*n* = 20) to compare MD and PSD before and after SLT treatment. The outcomes are summarized in Table 5. Mean HVF MD was significantly higher at 5-year (−7.64 ± 6.57 dB) compared to pre-SLT (−5.61 ± 3.90 dB; *P* = 0.02) with a difference of −2.03 dB over 5-year. PSD was significantly increased at 3-year (5.30 ± 2.91 dB) and 5-year (6.84 ± 2.62 dB), compared to pre-SLT (4.63 ± 2.70 dB; *P* = 0.04 and ≤0.01, respectively) (Table 5).

**TABLE 5:** Humphrey visual field parameters in eyes that did not receive subsequent surgical intervention.

		Mean (dB)*	SD	*P*-value^†^
HVF MD	Pre-SLT	−5.61	3.90	–
	3-year	−6.84	5.84	0.05
	5-year	−7.64	6.57	**0.02**
HVF PSD	Pre-SLT	4.63	2.70	–
	3-year	5.30	2.91	**0.04**
	5-year	6.84	2.62	**≤0.01**

*Twenty eyes that did not receive subsequent surgical intervention in the 5-year follow-up period were used in this analysis. Two eyes were missing one or more HVF. Only one eye from each patient was used in this analysis. ^†^*P*-values refer to comparison with pre-SLT values. Bold values indicate the *p*-value <0.05.

### 3.5. OCT parameters

The OCT parameters are summarized in Table 6 for the 19 eyes that did not receive a subsequent surgical intervention in the 5-year follow-up period and had complete OCT data. While average RNFL thickness and superior quadrant RNFL thickness were unchanged at 3-year and 5-year (both *P* > 0.05), the inferior quadrant RNFL thickness decreased significantly at 3-year (89.7 ± 20.5 μm) and 5-year (88.5 ± 19.3 μm), compared to pre-SLT RNFL thickness (94.0 ± 23.2 μm; both *P* ≤ 0.01, respectively) (Table 6).

**TABLE 6:** OCT parameters in eyes that did not receive subsequent surgical intervention.

RFNL thickness		Mean (μm)*	SD	*P*-value^†^
Mean	Pre-SLT	74.6	13.2	–
	3-year	73.3	11.8	0.21
	5-year	72.5	11.8	0.10
Superior	Pre-SLT	89.8	20.6	–
	3-year	87.7	20.0	0.28
	5-year	85.9	19.3	0.12
Inferior	Pre-SLT	94.0	23.2	–
	3-year	89.7	20.5	**≤0.01**
	5-year	88.5	19.3	**≤0.01**

*Nineteen eyes that did not receive subsequent surgical intervention in the 5-year follow-up period were used in this analysis. Three eyes were missing one or more OCT. Only one eye from each patient was used in this analysis. ^†^*P*-values refer to comparison with pre-SLT values. Bold values indicate the *p*-value <0.05.

## 4. Discussion

In this study, we examined the long-term outcomes of adjuvant SLT treatment on glaucomatous eyes that were receiving medical therapy. One of our main findings was that we found that a large percentage (49%) of eyes received a subsequent surgical intervention within the 5-year follow-up period after SLT treatment. Another main finding was that although IOP was significantly lower than baseline after 5 years in the eyes that did not receive a subsequent surgical intervention, the number of glaucoma medications was significantly increased, the HVF MD and PSD were significantly more deviated, and the inferior quadrant RNFL thickness was significantly decreased at 5 years compared to pre-SLT. Finally, we also found that patient age ≥ 69 years was significantly associated with increased risk of receiving subsequent surgical procedure to control IOP over the 5-year period after SLT treatment.

Although SLT has been shown to provide significant IOP reduction for glaucomatous eyes receiving concurrent medical therapy during the short-term follow-up period (10, 11, 19–21), we found that 49% of eyes received a subsequent surgical intervention in addition to continued medical therapy within 5 years to control IOP in response to progressively decreasing visual function. For the other 51% of eyes, one-time SLT with continued medical therapy significantly lowered IOP without subsequent surgical intervention. However, this group had a significant increase in the number of glaucoma medications (2.7 ± 1.6 at 5-year compared to 2.0 ± 1.1 before SLT) (Table 4). These data suggest that SLT may have some value as an adjuvant therapy for glaucomatous patients in lowering IOP; however, additional medical management (e.g., increased number of glaucoma medications) may be necessary to continue to observe the IOP-lowering effects of SLT at 5 years following treatment.

In comparison to previous studies that looked at the long-term effects of SLT on glaucomatous eyes, our study had a similar or lower rate of subsequent surgical intervention. One retrospective chart review study of 90° SLT had a low success rate of 24% after 4 years, where failure was defined as having repeat SLT, change in medical treatment, or other surgery (22). Our study looked at eyes receiving 180°, so we cannot directly compare with this study. However, previous studies suggest that 180° SLT is more efficacious at lowering IOP than 90° SLT after 1 year (20, 23). Other studies using 180° SLT showed low survival rates of 25% after 5 years, with survival being defined as at least 20% lowering of IOP with no additional surgical or medical intervention (24). In our study, we specifically examined whether eyes received additional surgical or laser intervention and excluded an increase in medical therapy from our survival analysis, which may explain our higher cumulative survival rate of 51% at 5 years. In another long-term study following early glaucoma patients treated with first line SLT, the re-treatment rate for receiving a repeat SLT was 60% over 10 years, which was slightly higher than ours of 49% over 5 years (14). Nonetheless, our data agree with previous studies that the rate of having a subsequent intervention is nearly half of patients receiving SLT over long-term follow-up.

Many previous studies have examined predictors of success of SLT. In this study, we examined factors that may be associated with having a subsequent surgical intervention by calculating hazard ratios over the 5-year follow-up period. We found that older age was significantly associated with greater risk of receiving a subsequent surgical intervention within 5-year (Table 3). A previous study reported that higher baseline IOP was associated with increased risk of re-treatment with 360° SLT within the 10-year follow-up period (14). In our study, baseline IOP was not significantly associated with risk of subsequent surgical intervention (Table 3). This previous study also did not find an association between age at SLT and risk of re-treatment. One difference between this previous study and ours that could account for these different findings is that our study examined adjuvant use of 180° SLT in glaucoma patients with continued medical therapy, while the previous study looked at first-line 360° SLT in treatment-naïve early glaucoma patients. Another factor that may account for our findings is the relatively small sample size of 51 eyes in our study. Further study is needed to elucidate the factors associated with risk of further surgical treatment in eyes receiving adjuvant SLT with continued medical therapy.

One of the strengths of this study is that we examined functional assessments (HVF and OCT) over the follow-up period, whereas previous long-term studies have not thoroughly examined such parameters. We found significant decreases in the functional assessments (HVF and OCT) over the 5-year follow-up period, specifically in patients who did not receive a subsequent surgical intervention. We had 20 eyes from 20 patients who did not receive a subsequent surgical intervention with complete HVF data, and we found mean MD declined from −5.61 to −7.64 dB over 5-year, or −0.41 dB per year if consistent rate of decline. One previous retrospective study examined early POAG patients receiving first-line SLT and found a steep decline in MD on HVF during years 6–10 of follow-up but only a slight decline over the first 6 years of follow-up, with a mean visual field MD change of −0.2 dB per year (14). The authors stated that this could have been due to the significant drop-out rate of patients over the follow-up period; the study started with 105 patients and only had 18 patients by the 10-year follow-up. The difference between our rates of MD decline is likely due to different patient populations; we examined glaucomatous eyes receiving medical therapy and adjuvant SLT in a mostly black/African American population, whereas Ansari’s study examined early glaucomatous eyes receiving first-line SLT in an unspecified patient population in the United Kingdom. We additionally examined the PSD and found a significant increase in PSD at 3- and 5-year compared to pre-SLT. These data suggest that HVF parameters may still decline, despite having significantly lower IOP and not receiving a subsequent surgical intervention.

We also examined the RNFL *via* OCT and found a significant decrease in inferior quadrant RNFL thickness at 3- and 5-year compared to pre-SLT in our 19 eyes from 19 patients with complete OCT data of the eyes that did not receive a subsequent surgical intervention. Previous studies found no significant change in RNFL in the short-term follow-up period of 6 months to 1 year after SLT (25, 26). To our knowledge, no other long-term studies have examined RNFL thickness in glaucomatous eyes receiving adjuvant SLT with continued medical therapy. Our data suggest that even with significantly lower IOP at 5-year follow-up, patients may have continued decreasing visual function as observed through decreasing RNFL thickness in the inferior quadrant (Table 6). Our findings on HVF and OCT exams suggest that patients who do not receive a subsequent surgical intervention to manage IOP may still be having statistically significant functional decline even with continued medical management.

There were some limitations in our study. First, this study was retrospective, meaning that no specific management protocol was followed among surgeons at Boston Medical Center. Additionally, because this study was retrospective, not all patients had every HVF or OCT parameter, therefore, analysis was conducted with those who had sufficient data. Also, our study contained a limited number of eyes from single healthcare center. In order to capture more patients with SLT, we included five eyes with NTG and two with uveitic glaucoma, so not all of our patients had the same glaucoma diagnosis; however, we included these, because statistical outcomes were not different when these eyes were excluded from analysis. Lastly, while we defined survival as not receiving a subsequent surgical procedure to control IOP, there were factors that this metric did not capture, such as if the patient did not wish to have a subsequent procedure.

In summary, this study found that a large percentage (49%) of eyes received a subsequent surgical intervention within the 5-year follow-up period after SLT treatment, with higher risk associated with higher age at time of SLT. Unlike previous studies on the efficacy of adjuvant SLT, we examined RNFL and HVF parameters and found that for the eyes that did not receive further surgical treatment, there was progression of MD and PSD on HVF and inferior quadrant RNFL thinning on OCT at 5-year follow-up with a significant increase in number of glaucoma medications. Our findings suggest that SLT may have some long-term benefit to lowering IOP with continued medical therapy; however, a significant number of patients received subsequent laser/surgical procedures to lower and prevent worsening visual function. Overall, our study adds to the understanding of the long-term effectiveness of SLT.

## Data availability statement

The original contributions presented in this study are included in this article/supplementary material, further inquiries can be directed to the corresponding authors.

## Ethics statement

The studies involving human participants were reviewed and approved by Boston University Institutional Review Board (IRB: H-39121). Written informed consent for participation was not required for this study in accordance with the national legislation and the institutional requirements.

## Author contributions

BE-R: conceptualization, resources, and supervision. DLS: data collection, analysis, and writing original draft. Both authors: writing, reviewing, editing, and approve the submitted version.
